# Role of Hedgehog Signaling Pathway in NASH

**DOI:** 10.3390/ijms17060857

**Published:** 2016-06-01

**Authors:** Mariana Verdelho Machado, Anna Mae Diehl

**Affiliations:** 1 Division of Gastroenterology, Department of Medicine, Duke University Medical Center, Durham, NC 27710, USA; 2 Gastroenterology Department, Hospital de Santa Maria, Centro Hospitalar Lisboa Norte (CHLN), Lisboa 1649-035, Portugal

**Keywords:** nonalcoholic fatty liver disease, hedgehog pathway, wound-healing response

## Abstract

Non-alcoholic fatty liver disease (NAFLD) is the number one cause of chronic liver disease in the Western world. Although only a minority of patients will ultimately develop end-stage liver disease, it is not yet possible to efficiently predict who will progress and, most importantly, effective treatments are still unavailable. Better understanding of the pathophysiology of this disease is necessary to improve the clinical management of NAFLD patients. Epidemiological data indicate that NAFLD prognosis is determined by an individual’s response to lipotoxic injury, rather than either the severity of exposure to lipotoxins, or the intensity of liver injury. The liver responds to injury with a synchronized wound-healing response. When this response is abnormal, it leads to pathological scarring, resulting in progressive fibrosis and cirrhosis, rather than repair. The hedgehog pathway is a crucial player in the wound-healing response. In this review, we summarize the pre-clinical and clinical evidence, which demonstrate the role of hedgehog pathway dysregulation in NAFLD pathogenesis, and the preliminary data that place the hedgehog pathway as a potential target for the treatment of this disease.

## 1. Introduction

Nonalcoholic fatty liver disease (NAFLD), the ectopic accumulation of fat in the liver that is unrelated to excessive alcohol consumption, is the liver pandemic of our century. NAFLD affects roughly one billion subjects worldwide [1]. When steatosis is accompanied by cell death and inflammation it is dubbed nonalcoholic steatohepatitis (NASH). The main risk factors for NAFLD/NASH are obesity and its associated metabolic disorders, such as type 2 diabetes mellitus and the metabolic syndrome [2]. Energy surplus overcomes the reservoir capacity of the adipose tissue, leading to ectopic accumulation of fat in the cardiovascular system, the pancreas and the liver [3]. The majority of individuals affected with NAFLD have non-progressive, isolated steatosis; about a quarter will develop NASH, and fewer than 10% will progress to liver cirrhosis and end-stage liver disease [4]. However, due to the high prevalence of NAFLD, it is already the second cause of liver transplantation in the US [5], and the most rapidly growing cause of liver transplantation in patients with hepatocellular carcinoma [6]. These epidemiological data have huge implications for the management of NAFLD: To follow and/or treat all individuals with NAFLD would be impractical and pointless. On the other hand, we clearly need to identify those at risk for severe liver-related morbidity and mortality. Our aim should be to identify this high-risk subpopulation in an effective, non-invasive, simple, and inexpensive way. Ideally, we should also have an effective treatment to apply. Recent epidemiological studies have demonstrated that neither the severity of steatosis, nor the presence of hepatocellular injury (*i.e.*, NASH), independently predict which NAFLD patients will develop bad liver outcomes [7,8,9]. On the other hand, NAFLD prognosis strongly correlates with the presence and severity of liver fibrosis [7,8]. Liver fibrosis is a manifestation of defective regeneration and thus, whether or not liver injury is repaired effectively is a better determinant of liver outcome than the severity of the insult (steatosis), or the severity of the injury (hepatocellular ballooning and NASH), *per se*. Lipotoxic insults that damage the liver trigger a wound-healing response to regenerate normal hepatic architecture and function. This process involves coordinated actions of different cell types, such as epithelial cells, progenitor cells, matrix-producing cells, endothelial cells and inflammatory cells, which collaborate to restrain toxicity and match the increased metabolic demands required to remodel the matrix, replace lost liver cells, and regenerate functional liver mass. Inability to assemble a wound-healing response may lead to liver failure. However, an overly exuberant response leads to excessive fibrogenesis and promotes scarring that may progress to cirrhosis and its complications. In fact, a study evaluating hepatic gene expression in patients with NAFLD showed that the most important difference between patients with mild NAFLD and NAFLD with advanced fibrosis was up-regulation of several genes in tissue repair and regeneration [10]. Therefore, understanding the mechanisms governing the wound-healing response is critical to develop therapeutic strategies that optimize liver repair to permit full recovery from fatty liver damage. The hedgehog pathway is a pivotal maestro of the wound-healing response, and its actions are conserved across different organs, including the skin [11], lung [12], kidney [13], pancreas [14] and liver [15]. Because hedgehog is the best characterized pathway that mediates liver fibrosis in NAFLD, we will summarize the role of hedgehog in the pathogenesis and progression of NAFLD, in this review.

## 2. The Hedgehog Signaling Pathway

The hedgehog (Hh) pathway was first identified by Nüsslein-Volhard and Wirschaus, in a genetic screen in *Drosophila melanogaster* [16]. Flies deficient in Hh had developmental defects in the cuticle, displaying a layer of disorganized hair-liked bristles that resembled the mammal hedgehog. Hh is a morphogen, and as such, its effect on cell fate depends on its local concentration. Hh diffuses to the extracellular matrix and thus, cells closer to the Hh-producing cells are exposed to high concentrations of Hh ligands [17]. Hh ligands (Sonic hedgehog, Shh; Indian hedgehog, Ihh; and Desert hedgehog, Dhh) are produced as 45 kDa precursor proteins, and undergo autocatalytic cleavage. The resultant N-terminal fragment has intrinsic cholesterol transferase activity, which promotes cholesterol lipidation of the active N-terminal fragment. Cholesterol modification is very important for Hh activity, promoting its retention in plasma membrane lipid rafts where Hh ligands interact with other lipids. A member of the membrane-bound O-acyltransferase (MBOAT) protein family, skinny hedgehog (SKI), mediates a second lipidation with palmitic acid. Palmitoylation is necessary for full ligand activity, as well as for long-distance movement [18]. Release of Hh from producing cells occurs in one of three ways: a process facilitated by the protein Dispatched, through assembly in very low-density lipoproteins (VLDL), or through exosomes [18].

All three mammalian Hh ligands have similar affinity for Hh binding proteins. They are equipotent in some but not all cell types, denoting overlap but also some specificity in their action [19]. Shh and Ihh are expressed widely, though Shh is the predominant ligand in the proximal gut, and Ihh in the hindgut. Dhh expression, however, is restricted to the nervous tissue and testis [20].

The cellular receptor for Hh is the 12-transmembranar protein Patched (Ptch). Ptch exists in two isoforms: Ptch-1, which is the one definitely involved in the activation of the Hh pathway, and Ptch-2, which seems to be expressed independently of pathway activity [21]. Three co-receptors enhance ligand-receptor interaction: CAM-related down-regulated by oncogenes (Cdo), brother of Cdo (Boc), and growth arrest-specific (GAS)-1 [17].

Cells in the resting state express Ptch that exerts a repressing effect on Smoothened (Smo). When Hh ligand binds to Ptch, it eliminates the repressing effect on Smo, allowing activation of the hedgehog pathway, through regulation of the processing and stability of Gli transcription factors. In short, when Smo is inactive, Gli factors are either degraded or processed in inactive forms. In contrast, when Smo is active, full-length Gli factors (or processed active forms) are stabilized and can accumulate/translocate to the nucleus, where they act as transcription factors.

In the absence of Hh ligand, Gli couples to a suppressor protein complex composed by fused kinase (Fu), suppressor of Fused (Sufu) and Costal-2 (Cos) [20,22]. This complex sequesters Gli in the cytoplasm promoting its sequential serine phosphorylations by protein kinase A (PKA), glycogen synthase kinase (GSK)-3β, and members of casein kinase-1 (CK1) family. Phosphorylation enhances binding of Gli to β-transducin repeat-containing protein (βTrCp), which targets Gli for ubiquitination and subsequent proteasome degradation. Partial degradation generates an inhibitor Gli-peptide that can translocate to the nucleus and repress transcription. Active Smo allows dissociation of Sufu from Gli [23]. Full-length Gli-protein can then translocate to the nucleus, where it acts as a transcription factor. Important known target genes are: vascular endothelial growth factor (VEGF), angiopoietin-1 and -2 (in endothelial cells); snail, twist-2, FoxF1, α-smooth muscle actin (α-SMA), vimentin, interleukin (IL)-6 (in fibroblasts/myofibroblasts); and Sox-2, Sox-9 and Nanog (in stem/progenitor cells) [20].

Gli proteins belong to the Kruppel-like family of transcription factors with highly conserved zinc finger DNA-binding domain [21]. Mammals have three Gli proteins: Gli-1, Gli-2 and Gli-3, which behave differently. Gli-1 and Gli-2 transcription profiles overlap, but are not identical [21]. Unlike the other Gli factors, Gli-1 is not proteolytically processed to a repressor form. Gli-1 is also a direct transcriptional target of Gli-2 [24]. Gli-3 acts mainly as a transcription repressor, with very efficient proteolytic processing, whereas Gli-2 acts mainly as a transcription activator, with an extremely inefficient proteolytic processing [25].

The activation of Hh signaling through Smo seems to require the presence of primary cilia. Primary cilia are small, immotile cilia, elaborated in interphase by most quiescent, differentiated cells [26]. Primary cilia are made of polymerized tubulin, and consist of the basal body (that derives from the mother centriole at the end of cell division), and the filamentous axoneme that protrudes into the extracellular space.

**Figure 1:**
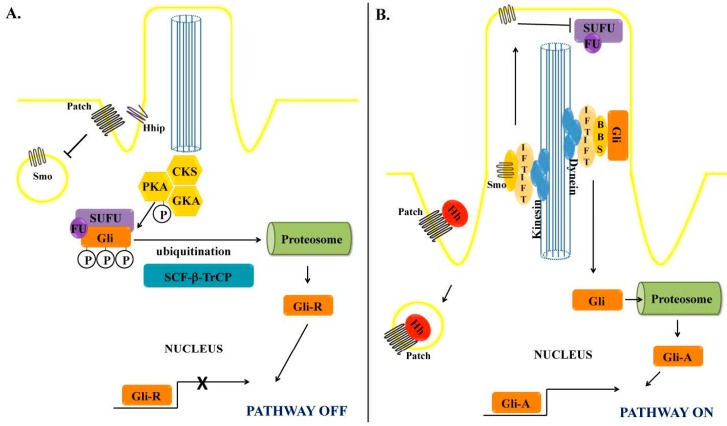
Hedgehog signaling pathway and the primary cilia. (**A**) In the absence of Hedgehog (Hh) ligand, Gli localizes in the cytoplasm as part of an inhibitory complex with Fused kinase (Fu) and Suppressor of Fused (SuFu), which allows the sequential phosphorylation by several kinases: Protein kinase A (PKA), glycogen synthase-3β (GSK3β) and casein kinase-1 (CK1). Thereafter, ubiquitination by Skip-Cullin-F-box (SCF) protein/β-Transducing repeat Containing Protein (TrCP) primes the phosphorylated Gli to limited proteosomic degradation, exposing the N-terminal repressor domain (GliR), which translocates to the nucleus and represses; (**B**) When Hh ligand binds to Ptch, it releases the inhibitory effect of Ptch on Smo that localizes in cytoplasmic vesicles. Smo then undergoes anterograde movement along the cilia, directed by kinesin and facilitated by the ciliary proteins Bardet-Biedl syndrome proteins (BBS) and intraflagellar transport proteins (IFP). At the tip of the cilia, Smo releases Gli from the suppressor complex, allowing it to move along the cilia, directed by dynein proteins. Unphosphorylated Gli undergoes limited proteosomal degradation, exposing the C-terminal activator domain (GliA), which translocates to the nucleus promoting gene transcription.

In resting cells, Smo resides in intracytoplasmic vesicles outside of the primary cilia. Hh binding removes Ptch from the primary cilia, allowing Smo to accumulate in the cilia membrane. Smo can then move along the cilia from the base to the tip, in a kinesin motor protein-based transport system, which is facilitated by the ciliary Bardet-Biedl syndrome proteins (BBS) and intraflagellar transport proteins (IFP). At the tip of the cilia, Smo enables removal of Gli from the inhibitor complex with Sufu. Free Gli then moves along the cilia in a retrograde fashion via a dynein motor protein-based transport system, which is facilitated by BBS, IFP and Kif7. Full length Gli ultimately translocates from the cytoplasm to the nucleus, where it acts as a transcription factor [20] (Figure 1).

The Hh pathway has several intrinsic mechanisms of negative regulation that limit sustained activation. For example, Gli, the main effector in the Hh pathway, increases the expression of important inhibitors of the pathway. In fact, three direct Gli-target genes are Ptch, hedgehog-interacting protein (Hip) and Foxa2, all of them can inhibit Hh pathway activity. Ptch constitutively suppresses Smo, Hip binds to Hh and prevents ligand from engaging Ptch so that Smo cannot be de-repressed; and Foxa2 suppresses Gli-2 transcription, thereby depleting cells of the factor that drives transcription of Gli-1, the main activator of Hh target gene expression [27].

In addition to the aforementioned “canonical” Hh signaling pathway, two types of non-canonical Hh signaling have been described: type 1 is Ptch-dependent (but Smo-independent) and type 2 is Smo-dependent (but does not require Hh interaction with Ptch) [21,22]. In type 1 signaling, binding of Hh ligand to Ptch prevents Ptch from directly interacting with, and activating, caspases [28], and thus has an anti-apoptotic effect. In addition, the interaction promotes proliferation by preventing Ptch from blocking cyclin B translocation into the nucleus [29,30]. In type 2 signaling, Smo behaves as a 7-transmembrane protein that has a G-protein-coupled receptor (GPCR)-like function and acts independently of Gli and of the primary cilia [31]. The GPCR-like functions of Smo engage a calcium-AMP kinase axis that induces a Warburg-like glycolytic metabolic reprogramming in muscle and adipose tissue [32]. Smo GPCR-like activity also stimulates small GTPases that promote cytoskeletal rearrangement allowing migration of fibroblasts, and tubulogenesis in endothelial cells [33,34,35].

Finally, Gli-2 transcription/activation can be induced by Hedgehog-ligand independent pathways, including transforming growth factor (TGF)-β, phosphatydilinositol 3-kinase (PI3K)/AKT, Ras and mitogen-activated protein kinases (MAPK)/extracellular signal-regulated kinases (ERK) [22]. Osteopontin, besides being a target gene of Gli, also inhibits GSK3β, thereby promoting Gli activation [36].

## 3. Hedgehog Pathway and the Wound Healing Response

The Hh pathway is a recognized maestro of the wound healing response [37]. The wound-healing response is a coordinated reaction to liver injury that aims to overcome the loss of hepatic structure and function that results when liver cells die. Injured or fatty hepatocytes cannot mount an adequate proliferative response to replace these cells [38], and hence progenitor cells are crucial for sick livers to regenerate. Progenitors in the liver (similar to other populations of stem/progenitor cells [39]) are sensitive to Hh [40,41,42,43]. Indeed, Hh activation enhances progenitor cell viability and proliferation, whereas Hh inhibition promotes progenitor differentiation or cell death by apoptosis [40,44]. Another conserved wound healing response that occurs after liver injury is the development of an inflammatory reaction, which is also strongly regulated by the Hh pathway. For example, hepatic NKT cells respond to Hh with improved viability and proliferation, and acquire a profibrogenic phenotype that includes up-regulating their expression of IL-13 [45]. Hh also directly induces M2 pro-fibrogenic polarization of macrophages/Kupffer cells, further crafting a pro-fibrogenic liver microenvironment [46]. Another important player in the wound healing response is the hepatic stellate cell (HSC), the main source of myofibroblasts in the liver [47]. HSC not only produce the extracellular matrix necessary to maintain hepatic architecture during injury, they are a rich source of paracrine trophic substances that act on all other cell types involved in the healing response [37], and have recently been shown to function as progenitor cells themselves [48]. Excessive HSC activation may lead to anomalous matrix deposition that causes progressive fibrosis. Hh enhances HSC survival by inhibiting apoptosis, promotes HSC proliferation, and stimulates HSC to undergo an epithelial to mesenchymal-like transition in order to acquire a myofibroblastic phenotype [49]. Lastly, liver sinusoidal endothelial cells respond to Hh with capillarisation of hepatic sinusoids and vascular remodeling; perpetuation of this response favors the development of portal hypertension [50].

Whereas in the healthy liver the expression of Hh ligands is barely detected [40], Hh pathway activation increases proportionally to the severity and duration of the liver insult [42,51]. During injury, several cell types up-regulate expression of Hh ligands. For example, Hh production is virtually absent in healthy hepatocytes, but injured ballooned hepatocytes are a major source of Hh ligands in NAFLD [51,52,53]. Other sources of Hh ligands during a regenerative/repair response in the liver are inflammatory cells [45,46], activated ductular/progenitor cells [54] and HSC [49,55,56].

Although the hedgehog pathway seems important in wound-healing response/regeneration in different systems besides the liver, such as kidney, skin, cardiovascular system [57], a recent report in the lung showed that the hedgehog pathway may be important in maintaining adult lung quiescence and is down-regulated in response to epithelial injury [58]. These data demonstrate how complex this exciting pathway is, and further research is needed to clarify its function in liver health and repair.

**Figure 2:**
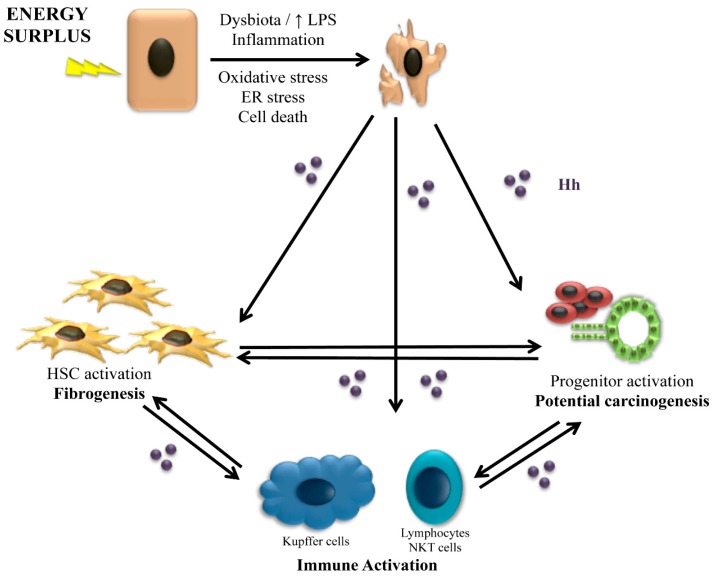
The role of Hedgehog on the wound-healing response. Energy surplus leads to fat accumulation in the hepatocytes, which promote oxidative stress, endoplasmic reticulum (ER) stress and cell death. The injury of hepatocytes is promoted by an inflammatory state, among other factors, favored by a deregulated gut microbiota and increase in lipopolysaccharide (LPS). Injured and dying hepatocytes release hedgehog ligands (Hh) that act on the immune system increasing inflammation, in stellate cells and progenitors cells activating them and inducing fibrogenesis and pathways of hepatocarcinogenesis. Once started, the regenerative/repair response perpetuates through crosstalk between the different cell types involved.

In summary, the wound-healing response depends on coordinated cross-talk among different cell types. Injured hepatocytes produce Hh ligands that attract and activate inflammatory cells. Infiltrating inflammatory cells, in turn, up-regulate their expression of Hh ligands and begin to produce profibrogenic cytokines, such as IL-13 and transforming growth factor (TGF)-β. These factors, not only activate myofibroblasts, but also are toxic to hepatocytes, further increasing hepatocyte injury and Hh ligand production [43]. Hh ligands also activate progenitor cells, inducing a ductular reaction. Activated ductular/progenitor cells up-regulate expression of chemokines/cytokines such as CXCL16 and platelet-derived growth factor (PDGF), which recruit more inflammatory cells and promote accumulation of myofibroblasts [59,60]. Hh ligands also activate HSC, causing their transdifferentiation into myofibroblasts and thus, promoting a fibrogenic response. If this initially adaptive response is not appropriately constrained, excessive activation of HSC/myofibroblasts promotes progressive fibrosis, and excessive proliferation of relatively immature liver epithelial cells represses regeneration of fully functional hepatocytes, leading to liver failure and carcinogenesis (Figure 2).

## 4. The Role of Hedgehog in Animal Models of NASH

Activation of the Hh pathway is a conserved feature of chronic liver disease, and NAFLD/NASH is no exception. Different rodent animal models of NAFLD show activation of the Hh pathway, demonstrated by increased expression of Hh ligands and Hh-producing cells, with accumulation of nuclear Gli-2 positive cells and increased expression of Gli-target genes such as osteopontin [42,53,61,62,63,64,65]. Furthermore, the activation of the Hh pathway is proportional to liver injury, namely to hepatocyte injury/apoptosis, ductular reaction and, most importantly, fibrosis [42,53,65].

Lipotoxic dying hepatocytes are a main source of Hh ligands that can trigger the repair response during NAFLD/NASH. *In vitro* models of lipotoxicity demonstrated up-regulation of Hh ligands in hepatocytes incubated with saturated fatty acids and lysophospholipid [65,66]. The mechanism leading to Hh ligand expression has not been clearly demonstrated. However, agents that can induce endoplasmic reticulum stress or activation of the NFkB pathway mimic the lipotoxic phenotype [52,67].

In animal models of NASH, the Hh-responsive progenitor population expands, and Hh-stimulated HSC undergo epithelial-to-mesenchymal transdifferentiation into myofibroblasts acquiring a pro-fibrogenic phenotype [42,61]. Activated ductular progenitor cells and myofibroblasts, in turn, up-regulate their production of Hh ligands, and release pro-inflammatory and chemotactic cytokines, such as osteopontin and CXCL-16 [60,63]. Immune cells are recruited, namely NKT cells, which have a pivotal role in NASH pathogenesis. Active NKT cells, in its turn, secrete more Hh ligands and profibrogenic cytokines, such as IL-13, perpetuating the disease progression [62,68].

Genetically modified mice, with heterozygous deficiency of Ptch (Ptch^+/−^), which display an overly active Hh pathway, develop worse liver disease when submitted to a NASH-inducing diet [61,62,63]. In contrast, genetically modified mice with conditional liver-specific inhibition of Smo, were protected from liver injury and liver fibrosis in different dietary models of NASH, despite similar accumulation of ectopic fat in the liver [37,69]. A recent study took advantage of a transgenic mouse with transposon encoding Shh hydrodinamically delivered to the liver to extend knowledge about hedgehog’s role in NASH progression. Although this approach achieve expression of Shh in only 2%–5% of hepatocytes, it was sufficient to induce spontaneous liver fibrosis after 6 months and hepatocellular carcinoma after 13 months [70]. Hh ligands stimulate and increase proliferation of progenitor cells, as well as immune cells and hepatic stellate cells. As such, ductular progenitor cells, immune and hepatic stellate cells are Gli-2-positive (*i.e.*, Hh-responsive). Remarkably, 30%–50% of hepatocytes also exhibited nuclear Gli-2 expression. This finding challenges current dogma in the field, which posits that healthy hepatocytes cannot respond to Hh because they do not express primary cilia.

Different laboratories, studying different rodent models of diet-induced NASH, showed that pharmacological inhibition of Smo (vismodebig or LDE225) decreased activation of hedgehog pathway and consistently improved liver inflammation and fibrosis [61,69,71]. Those results place the Hh pathway as a potential therapeutic target in NASH.

## 5. The Hegdehog Pathway in Human NASH

The prevalence of human NAFLD is increasing worldwide in association with globalization of western lifestyles characterized by physical inactivity and overfeeding with predilection to sugar and fat enriched food. Roughly one fourth of the U.S. population has hepatic steatosis, however only a minority (2%–5%) will progress to NAFLD-related liver cirrhosis and end-stage liver disease [4]. Importantly, we still lack an effective treatment for this disease, which explains why NASH-related cirrhosis has become the second leading cause for liver transplantation in the US [5]. Liver prognosis is dictated by the fidelity of the wound healing response, with deregulated wound-healing promoting development of progressive fibrosis [7,8]. Hh is a crucial factor involved in this abnormal response to injury. Not only is Hh the best characterized fibrogenic pathway in animal models of NASH, but there is also strong human data that highlight its role in the pathogenesis of human cirrhosis.

Although isolated steatosis does not stimulate Hh pathway activation, steatohepatitis-related hepatocyte injury triggers Hh ligand production, and in human NASH the intensity of activation of the Hh pathway parallels the severity of liver disease. Hh pathway activity has been demonstrated to correlate with portal inflammation, hepatocellular ballooning, and markers of liver repair (e.g., numbers of hepatic progenitor cells and myofibroblasts) in NAFLD patients. More importantly, Hh activation correlates with the severity of fibrosis [51,61]. The major source of Hh ligands seems to be injured ballooned hepatocytes. In fact, the number of Shh expressing ballooned hepatocytes strongly correlates with fibrosis severity [51,72]. Furthermore, the number of Shh expressing ballooned hepatocytes also correlates with the severity of the ductular reaction, which strongly associates with fibrogenesis and carcinogenesis [73,74].

In the pediatric population, NAFLD can occur with a similar histology as in adults, or it can present a unique histology that is characterized by less hepatocellular ballooning but a predominantly portal phenotype, *i.e.*, intense ductular proliferation, portal inflammation and fibrosis. A tremendous increase in the number of portal Gli-2 positive cells has been demonstrated in this pediatric pattern of NASH [75] and it occurs most often in pre-pubertal children, paralleling the kinetics of hepatic Hh expression, which is high in children and falls after adolescence [76].

Recently, a *post hoc* evaluation of the PIVENS (Pioglitazone, Vitamin E for Non-alcoholic Steatohepatitis) trial, analyzed pre- and post-treatment liver biopsies from 30 patients randomized to vitamin E and 29 to placebo [77]. Loss of Shh expressing hepatocytes strongly correlated with treatment response in terms of aminotransferases levels, hepatocyte ballooning, ductular reaction, presence of NASH and, most importantly, fibrosis stage [77]. This evidence linking reduced Hh activity with improvement of NASH in humans complements and extends the aforementioned work in preclinical models which showed that pharmacological strategies that directly decreased Hh activity abrogated NASH progression.

The roles of canonical and non-canonical pathways in liver disease in general and NASH in particular is still a matter of debate. Whereas progenitor cells clearly express primary cilia and thus can engage the canonical Hh pathway, it has been suggested that HSC, immune cells and hepatocytes do not express primary cilia, and hence Gli-2 activation/Gli-1 expression would be the result of non-canonical pathways [78,79]. In addition, type 2 non-canonical Smo-dependent RhoA/Rho kinase activation of HSC has been suggested to play a role in hepatic fibrogenesis [80]. Further research is needed to clarify the relevance of these different signaling cascades to better delineate a treatment strategy. To date, the most studied inhibitors of the Hh pathway *in vitro* and in animal models of NASH are cyclopamine and vismodegib, both strong Smo antagonists, which bind Smo and inhibit of its ciliary localization [81]. Interestingly, although HSC are sensitive to factors that induce non-canonical Hh pathway activation, they are also highly responsive to Hh ligands, antibodies against Hh and to both cyclopamine and vismodegib [49,55,56]. Furthermore, while healthy hepatocytes do not respond to cyclopamine, murine hepatocytes isolated after partial hepatectomy respond to cyclopamine with increased proliferation [82]. This suggests that the presence of a primary cilium may be a dynamic event, depending on the cell cycle phase and maybe in response to injury [83].

The aggregate data in animal models and human NASH strongly suggest that modulation of the Hh pathway may be a treatment for NASH that prevents fibrosis progression. As such, patients that would most benefit from treatment would be the ones that already have liver fibrosis to prevent progression to cirrhosis and its complications. This approach is particularly appealing because several Hh inhibitors have already been approved by the FDA to treat other diseases such as basal cell carcinoma [84] and, thus, the time lag between preclinical/clinical research and treatment of actual NASH patients should be short.

## 6. Conclusions

NASH-associated cirrhosis occurs when the liver reacts to lipotoxicity with a deregulated wound-healing response that is maladaptive. The liver must repair and regenerate when confronted with injury or death will ensue, just as Prometheus’ survival depended upon his liver’s ability to regenerate after being eaten by Zeus’ eagle. When the eagle repeatedly eats the liver or when the repair/regenerative response cannot be shut down even when the satiated eagle stops eating the liver, the protracted wound-healing response leads to progressive fibrosis and carcinogenesis. The Hh pathway is a known maestro orchestrating an integrated regenerative response by the different cellular players involved in wound-healing. The Hh pathway is hibernating in the normal liver, but it wakens during injury, and the intensity of its activation is a reflection of the severity of liver injury. Data from animal models and human NASH have consistently confirmed that Hh pathway activation correlates with the severity of liver disease. More importantly, direct pharmacological inhibition of the Hh pathway prevents disease progression in different rodent models of NASH and Hh pathway activity decreases with improvement of NASH in humans. These findings position the Hh pathway as a potential therapeutic target in NASH, the hepatic pandemic of our century for which development of an effective treatment is a priority for hepatologists worldwide.

## Abbreviations

BBS	Bardet-Biedl syndrome proteins
Boc	brother of Cdo
Cdo	CAM-related downregulated by oncogenes
Cos	Costal-2
CK1	casein kinase-1
Dhh	Desert hedgehog
Fu	fused kinase
GAS-1	growth arrest-specific-1
GPCR	G-protein-coupled receptor
GSK	glycogen synthase kinase
Hh	hedgehog
Hip	hedgehog-interacting protein
HSC	hepatic stellate cell
IFP	intraflagellar transport proteins
Ihh	Indian hedgehog
IL	interleukin
MBOAT	membrane-bound O-acyltransferase
NAFLD	nonalcoholic fatty liver disease
NASH	nonalcoholic steatohepatitis
PDGF	platelet-derived growth factor
PKA	protein kinase A
Ptch	Ptched
Shh	Sonic hedgehog
SKI	skinny hedgehog
SMA	smooth muscle actin
Smo	smoothened
Sufu	suppressor of fused
TGF	transforming growth factor
TrCp	transducing repeat-containing protein
VEGF	vascular endothelial growth factor
VLDL	very low-density lipoproteins
